# Bioengineering of a tumour-stroma 3D-tumouroid co-culture model of hypopharyngeal cancer

**DOI:** 10.1242/bio.059949

**Published:** 2023-05-17

**Authors:** Santu Saha, Rachel Howarth, Sweta Sharma-Saha, Charles Kelly

**Affiliations:** ^1^ Translational and Clinical Research Institute, Newcastle University Centre for Cancer, Leukaemia Research Cytogenetics Group, Wolfson Childhood Cancer Research Centre, Level 6, Herschel Building, Brewery Lane, Faculty of Medical Sciences, Newcastle University, Newcastle upon Tyne, NE1 7RU, UK; ^2^ Translational and Clinical Research Institute, Newcastle University Centre for Cancer, Paul O'Gorman Building, Faculty of Medical Sciences, Newcastle University, Newcastle upon Tyne, NE2 4HH, UK; ^3^ Department of Clinical Oncology, Northern Centre for Cancer Care, Freeman Hospital, Newcastle upon Tyne, NE7 7DN, UK

**Keywords:** Head and neck cancer, Hypopharyngeal cancer, Tumour-stroma, 3D-tumouroid co-culture, Radiotherapy, Immunotherapy

## Abstract

Head and neck cancer (HNC) differs at anatomical sites and hypopharyngeal cancer (HPC) is a type of HNC. The non-surgical treatment option for advanced cases of HPC is radiotherapy (RT) with or without chemotherapy but survival is poor. Thus, new treatment approaches in combination with RT are essential. Yet, obtaining post-RT treated tumour specimens and lack of animal models with identical anatomical sites are the major translational research barriers. To overcome these barriers, for the first time, we have developed a tumour-stroma based *in vitro* three-dimensional (3D)-tumouroid co-culture model of HPC by growing FaDu and HS-5 cells together to mimic the complex tumour-microenvironment in a Petri dish. Before growing the cells together, imaging flow cytometry revealed distinct epithelial and non-epithelial characteristics of the cells. Growth rate of the 3D-tumouroid co-culture was significantly higher compared to the tumouroid monoculture of FaDu. Histology and morphometric analysis were done for the characterisation as well as the development of hypoxia was measured by CAIX immunostaining in this 3D-tumouroid co-culture. Taken together, this innovative *in vitro* 3D model of HPC resembles many features of the original tumour. The wider application of this pre-clinical research tool is in understanding newer combination (e.g. immunotherapy) treatment approaches with RT in HPC and beyond.

## Teaser

**Summary:** This paper describes an innovate tumour-stroma-based *in vitro* 3D tumouroid co-culture model of HPC developed by growing FaDu and HS-5 cells together to mimic the complex tumour microenvironment in a Petri dish. The wider application of this pre-clinical research tool is in understanding newer combination (e.g. immunotherapy) treatment approaches with radiotherapy in hypopharyngeal cancer and beyond.

## INTRODUCTION

Head and neck cancer (HNC) is the seventh most common cancer worldwide and differs in progression and outcome at differing anatomical sites (Mody et al., 2021). Hypopharyngeal cancer (HPC) is a type of HNC that appears at the inferior part of the pharynx (Board, 2021). In advanced cases (stages III–IV) of HPC, tumours reach more-than 4 cm in diameter and can spread to the larynx, oesophagus, lymph nodes and, thyroid gland (Board, 2021). This makes the treatment options very complex and challenges the functional restoration of the upper aerodigestive organs to give an acceptable quality of life (Eckel and Bradley, 2019). The non-surgical treatment option for HPC is radiotherapy (RT)±chemotherapy but the 5-year-survival rate is only approximately 44% (Eckel and Bradley, 2019). Thus, improving the RT-based treatment, which is the mainstay for advanced HPC, is an unmet clinical need and more laboratory-based research with RT is essential. However, obtaining post-RT-treated HPC tumour specimens is one of the major translational research barriers and to overcome this, new biological models are required. The use of immunosuppressed or transgenic animals with artificially suppressed or altered immunity for modelling specifically for HPC tumours at the same anatomical sites is another limitation and thus, *in vitro* models are indispensable (Bonartsev et al., 2022).

A tumour is a complex structure of many components that is called the tumour microenvironment and studying the effect of RT±anti-cancer drugs by culturing cancer cells at the flat surfaces is not always representative of the complex TME (Leek et al., 2016). Thus, to circumvent these barriers, we have developed a novel tool by growing HPC cells with immortal non-cancer bone stromal cells to mimic a tumour-like structure called the ‘three-dimensional (3D)-tumouroid co-culture’.

Stromal cells are an important component of the TME that promotes tumour growth by many mechanisms including immunosuppression (Turley et al., 2015). Immunotherapy along with RT is an emerging approach to treat HNC (Karam and Raben, 2019) but there have been few studies in the HPC variant (Eckel and Bradley, 2019). Within the TME, RT induces cell death in both tumour as well as stromal cell types (Berg and Pietras, 2022). The RT-induced cell death mediated inflammatory responses elicit complex immune responses (Portella and Scala, 2019) and our innovative 3D-tumouroid co-culture model can be exploited for studying this complex signalling *in vitro* to explore RT in combination with immunotherapy in HPC (Donlon et al., 2021).

Stromal cells consist of heterogeneous cell types. Therefore, it is potentially difficult for resourcing any single type of stromal cell. However, the commercially available bone marrow-derived HS-5 cell line is considered to be a robust tool for studying the immune and inflammatory responses (Adamo et al., 2020). Hence, in this study we have selected HS-5 cell line to create the stromal component of the TME and used FaDu cells to represent HPC tumours (Rangan, 1972). To our knowledge, combining these two cell types to establish tumour-stroma based 3D-tumouroid co-culture model of HPC has never been reported before.

Another important factor within the TME that can alter the outcome of RT is tumour hypoxia (Leek et al., 2016). Hypoxic HPC tumours are associated with poor survival after chemo-radiotherapy (CRT) (Bernstein et al., 2015) and despite this, to our knowledge there is no reported 3D *in vitro* hypoxic model of HPC for studying post-CRT molecular events.

To establish our 3D-tumouroid co-culture model, we have characterised two different cell types by immunostaining of epithelial and mesenchymal properties followed by morphometric analysis. We have compared the growth kinetics of the 3D-tumouroids grown as monoculture as well as in co-culture. Further, we have characterised the 3D-tumouroids based on their histology and conducted a time-lapse study to observe the progression of hypoxia. All these characterisations reveal that our 3D-tumouroid co-culture model closely resembles the original tumours obtained from the HPC patients in clinic.

## RESULTS

### Characterisation of the tumour (FaDu) and stromal (HS-5) cells

**Fig. 1.**
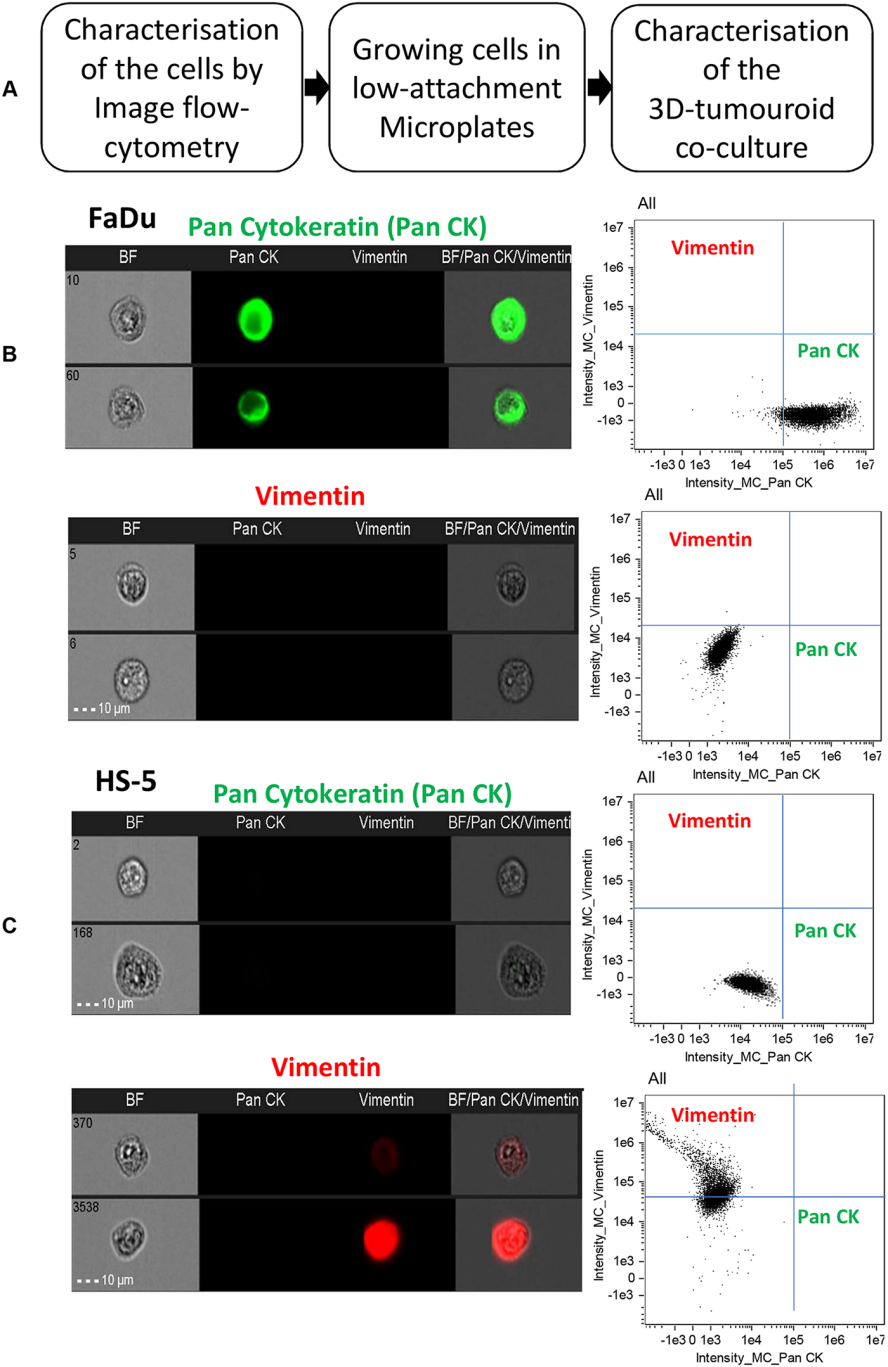
**Characterisation of the tumour (FaDu) and stromal (HS-5) cells by image flow cytometry.** (A) schematic presentation of the steps followed to establish the 3D-tumouroid co-culture model combining epithelial and stromal cell types. (B,C) FaDu and HS-5 cells were selected and characterised at first for their epithelial and mesenchymal features as a measure of the purity of these cells before taking them forward for growing in low-attachment microplates to establish the 3D-tumouroid co-culture model. For the characterisation of these two cell types, imaging flow cytometry was done staining the cells with Alexa Fluor 488 Anti-pan Cytokeratin (ab277270, Abcam) and Alexa Fluor 647 Anti-Vimentin (ab194719, Abcam) antibodies as epithelial and mesenchymal biomarkers. Images of 5000 cells from each of the staining conditions were captured on the ImageStreamX Mark II with fluorochrome excitation at 388 and 562 nm as well as brightfield illumination. Images of the objects were gated by plotting the aspect ratio of the objects against the area of the brightfield images to select round single objects. Images in focus were subsequently gated according to a threshold of the ‘gradient root mean square’ parameter. All in-focus round single cells were plotted in an XY plot of cytokeratin fluorescence intensity against vimentin fluorescence intensity to identify the relative degree of epithelial and mesenchymal phenotypes of each cell type. Image flow cytometry analysis confirmed the FaDu as epithelial and HS-5 as stromal cell types. Cells without any antibodies can be found in Fig. S1.

FaDu and HS-5 cells grown in monolayer were characterised at first by image flow cytometry and it was confirmed that FaDu are epithelial and HS-5 are stromal cell types (Fig. 1B,C). Pan-cytokeratin was used as the epithelial marker because of its wide use for the identification of tumours (Barak et al., 2004) and vimentin was used as the stromal cell marker (Ngan et al., 2007).

**Fig. 2.**
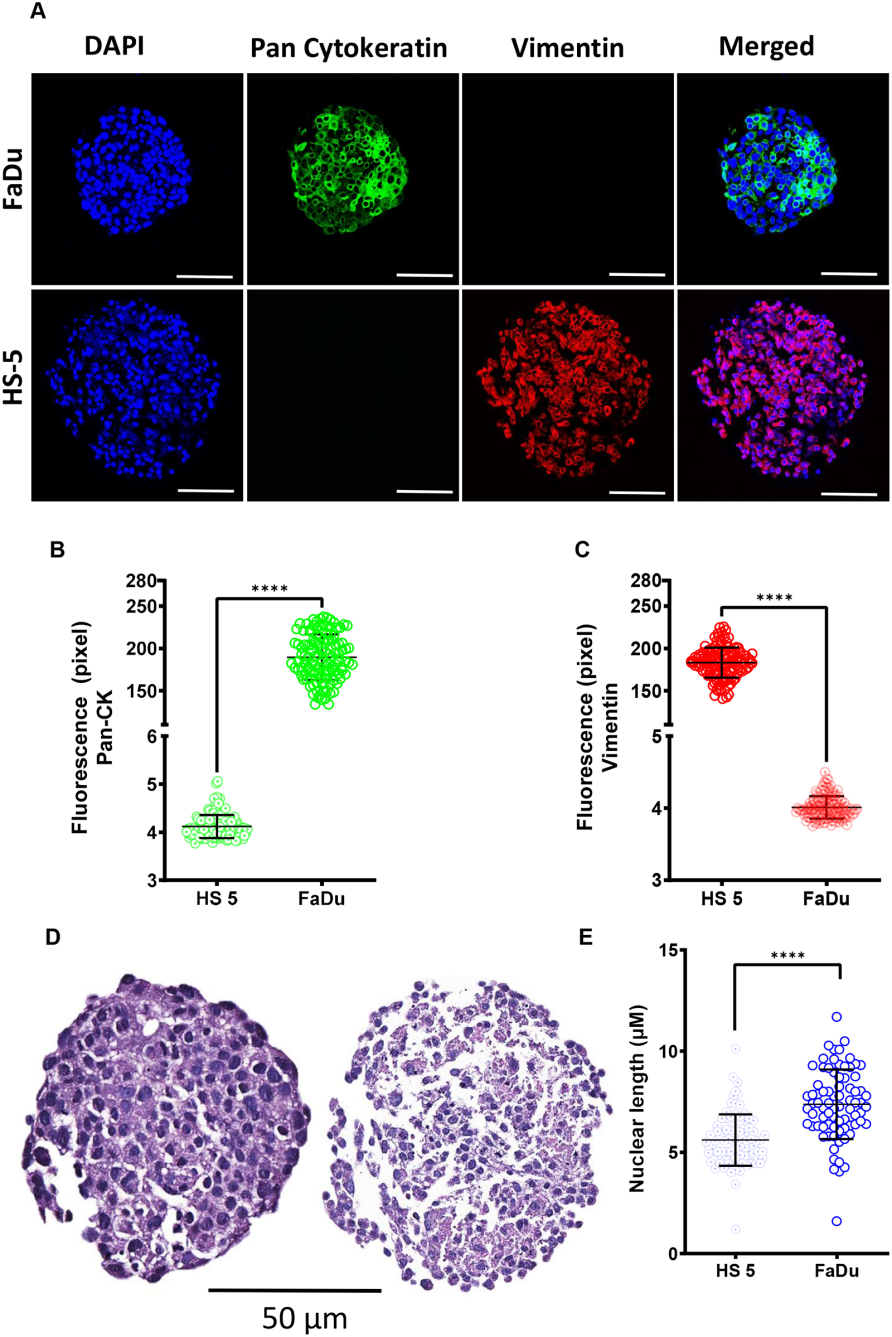
**Characterisation of the tumour (FaDu) and stromal (HS-5) cells at their multicellular structure as a 3D-tumouroid forms.** (A) FaDu (3000 cells were seeded and grown for 5 days) and HS-5 (6000 cells were seeded and grown for 10 days) cells were grown independently to test their potential for forming multicellular structures as 3D-tumouroids. To validate the image flow-cytometry data, the FFPE sections of these 3D-tumouroids were stained with Alexa Fluor 488 mouse anti-pan cytokeratin (1:500) and Alexa Fluor 647 rabbit anti-vimentin (1:500) antibodies together. Images were captured using Leica SPE confocal microscope under 10X objective with zoom factor 2. Scale bars: 20 µM. Composite images represent epithelial marker (pan-cytokeratin/green), non-epithelial/mesenchymal marker (vimentin/red) and nucleus (DAPI/blue). (B,C) Fluorescence intensities of the pan-cytokeratin and vimentin stained FFPE sections of the FaDu and HS-5 3D-tumouroids were measured using ImageJ software and data were analysed using Graphpad Prism 9.5.0 software. Groups were compared using unpaired two-tailed Welch's *t*-test. ****P<0.0001. (D) FFPE sections of the FaDu and HS-5 3D-tumouroids were also stained with PAS to see the differences in the sizes, shapes and morphology of the nucleus. Histology of the sections were scanned using Leica Aperio Image scanner. Nuclei stained dark blue. From the histology, two different cell types were clearly differentiated, and this helped us to identify these two different cell types present within the 3D-tumouroid co-culture when grown together and analysed further by the PAS staining given in Fig. 5. (E) Nuclear length (µM) of the PAS stained FaDu and HS-5 cells were measured using Leica Image Scope software and analysed using Graphpad Prism 9.5.0 software. Groups were compared using unpaired two-tailed Welch's *t*-test. ****P<0.0001.

**Fig. 3.**
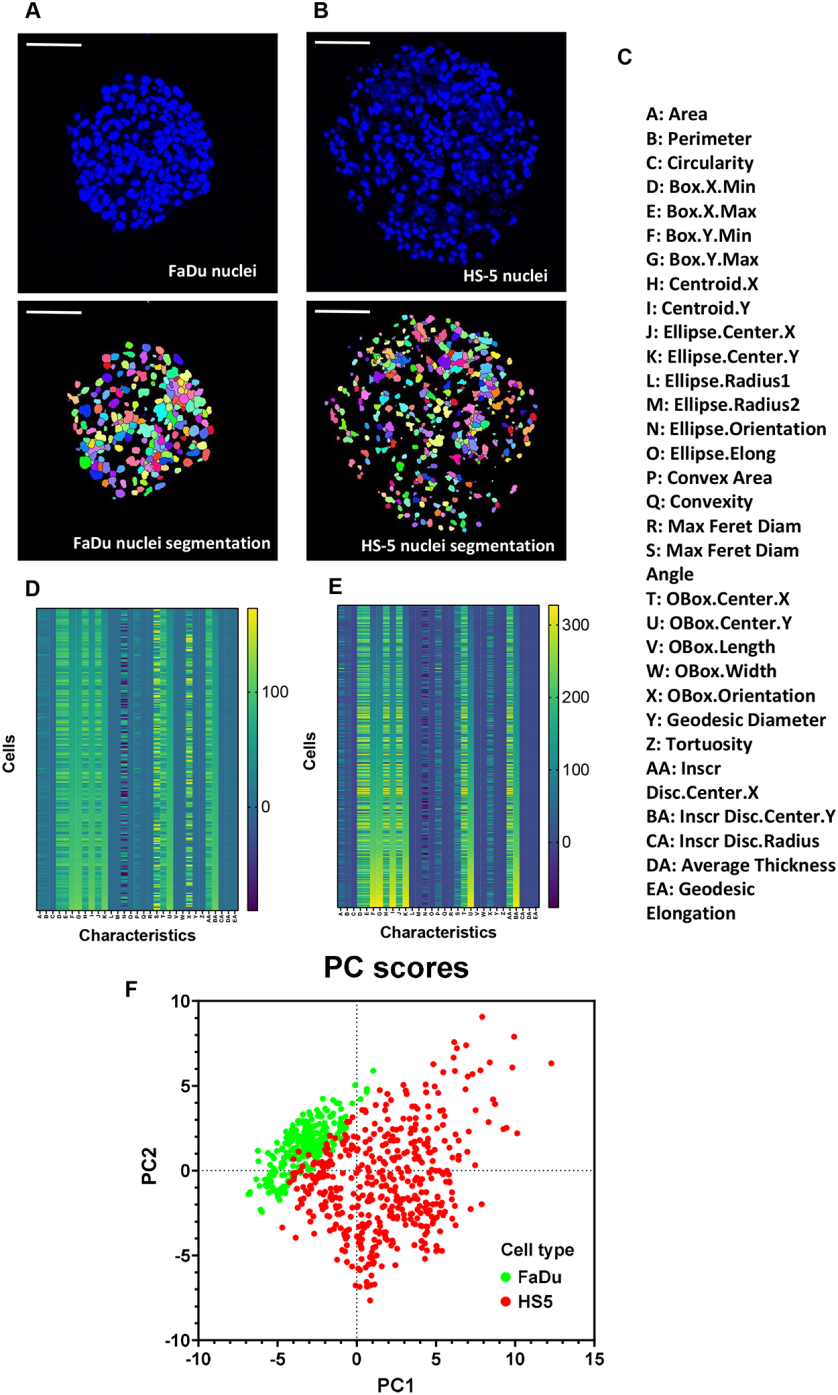
**Morphometric characterisation of the tumour (FaDu) and stromal (HS-5) cells at 3D-tumouroids.** (A,B) From the Fig. 2A, composite images of only DAPI stained sections of FaDu and HS-5 3D-tumouroids were obtained for the morphometric analysis to differentiate these two cell types. Nucles of these cells were segmented and individual nuclei segments were given a random pseudo-colour using MorphoLibJ plugin with ImageJ software. Scale bar: 20 µM. (C) Segmented nuclei were then characterised based on 31 different features available within the MorphoLibJ plugin of ImageJ software and presented in the form of heat map using Graphpad Prism 9.5.0 software: (D) FaDu and (E) HS-5 respectively. (F) For clarity, the 31 morphometric features were further analysed in a 2D graph by principal component analysis (PCA) using Graphpad Prism 9.5.0 software and from the PCA analysis two different population of cells i.e. FaDu (green) and HS-5 (red) could be distinctly identified. Eigenvectors obtained during the PCA analysis to select PC1 and PC2 at the X and Y axis respectively.

Next, these cells were tested for their potential for forming multicellular structures as 3D-tumouroids and image flow cytometry data was further validated by immunostaining of pan-cytokeratin and vimentin (Fig. 2A–C). Immunofluorescence intensity of pan-cytokeratin was significantly higher (∼50 fold, *P*<0.0001) in the 3D-tumouroid sections of FaDu than HS-5 (Fig. 2B). Likewise, the immunofluorescence and intensity of vimentin was significantly higher (∼50 fold, *P*<0.0001) in 3D-tumouroid sections of HS-5 than in FaDu (Fig. 2C). Thus, it was established that epithelial and stromal characteristics of these two cell types remain intact even in their 3D-multicellular form. Additionally, these two cell types were further characterised for their nuclear length (Fig. 2D,E) and morphometric features (Fig. 3A–F) before growing them together to develop the tumour-stroma based 3D-tumouroids co-culture model.

Histology of the 3D-tumouroid sections revealed there was a distinct difference between these two cell types as the nuclear length of FaDu cells were significantly larger compared to the HS-5 cells (∼1.5 fold, *P*<0.0001) (Fig. 2E). Immunostained 3D-tumouroid sections were further used for the morphometric analysis comparing 31 features (Fig. 3C). FaDu cells and HS-5 cells could be clearly differentiated using their morphometric features (Fig. 3D,E) and formed two distinct clusters using principal component analysis (PCA) (Fig. 3F).

### Growth kinetics and characterisation of the 3D-tumouroid co-culture

**Fig. 4.**
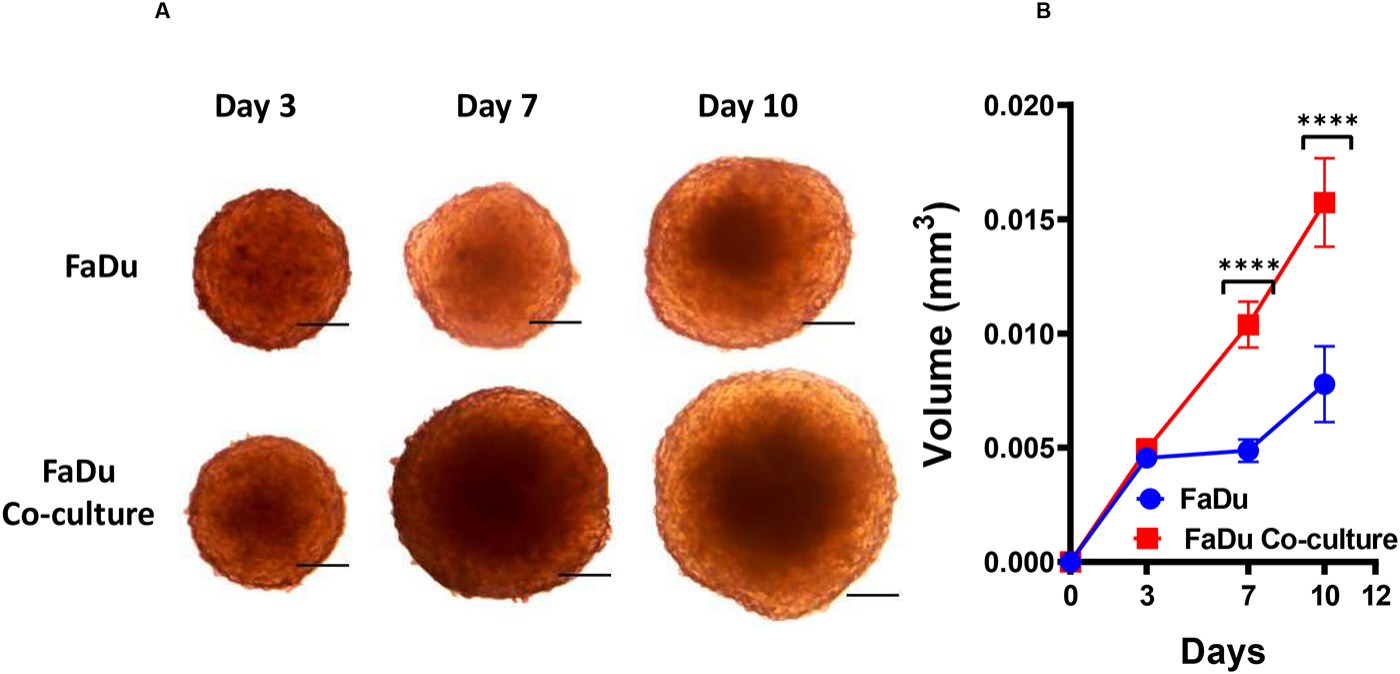
**Growth kinetics comparison between 3D-tumouroid mono and co-culture.** (A) Brightfield microscopic images (4X objectives) of the tumouroid monoculture (FaDu cells alone) and the tumouroid co-culture (FaDu plus HS-5 cells) at days 3, 7 and 10. Scale bar: 20 µM. (B) Brightfield microscopic images were analysed using ImageJ software for measuring the diameters of the tumouroids to analyse the tumouroid volume. Groups were compared in Graphpad Prism 9.5.0 software for unpaired two-tailed Welch's *t*-test. ****P<0.0001.

After the characterisation of FaDu and HS-5 cells individually at their two-dimensional (2D) and 3D culture conditions, these two cell types were allowed to grow together to test their ability of forming 3D-tumouroid co-culture. The 3D-tumouroid co-culture was successfully formed in the culture medium without any matrix and additives such as Matrigel (Fig. 4A). Growth of the 3D-tumouroid monoculture (FaDu cells alone) and the 3D-tumouroid co-culture (combining FaDu and HS-5 cells) were recorded at three different time points for a comparison between their growth kinetics (Fig. 4B). At day 3, the average volume of the 3D-tumouroid monoculture and the 3D-tumouroid co-culture was almost the same ∼0.005 mm^3^ (Fig. 4B). However, the difference in their growth kinetics were markedly noticeable on days 7 and 10 (Fig. 4B). On day 7 and 10, the average volume of the 3D-tumouroid co-culture was ∼0.010 mm^3^ and ∼0.015 mm^3^ respectively which was ∼twofold greater (*P*<0.0001) compared to the average volume of the 3D-tumouroid monoculture which were ∼0.005 mm^3^ and ∼0.0075 mm^3^ respectively (Fig. 4B). The growth kinetics of the 3D-tumouroid co-culture was also noticed to be exponentially increasing between day 3 to day 10 (Fig. 4B). Whereas, 3D-tumouroid monoculture growth on day 7 was almost same (∼0.005 mm^3^) as recorded on day 3 but increased ∼1.5 fold on day 10 (∼0.0075 mm^3^) (Fig. 4B).

**Fig. 5.**
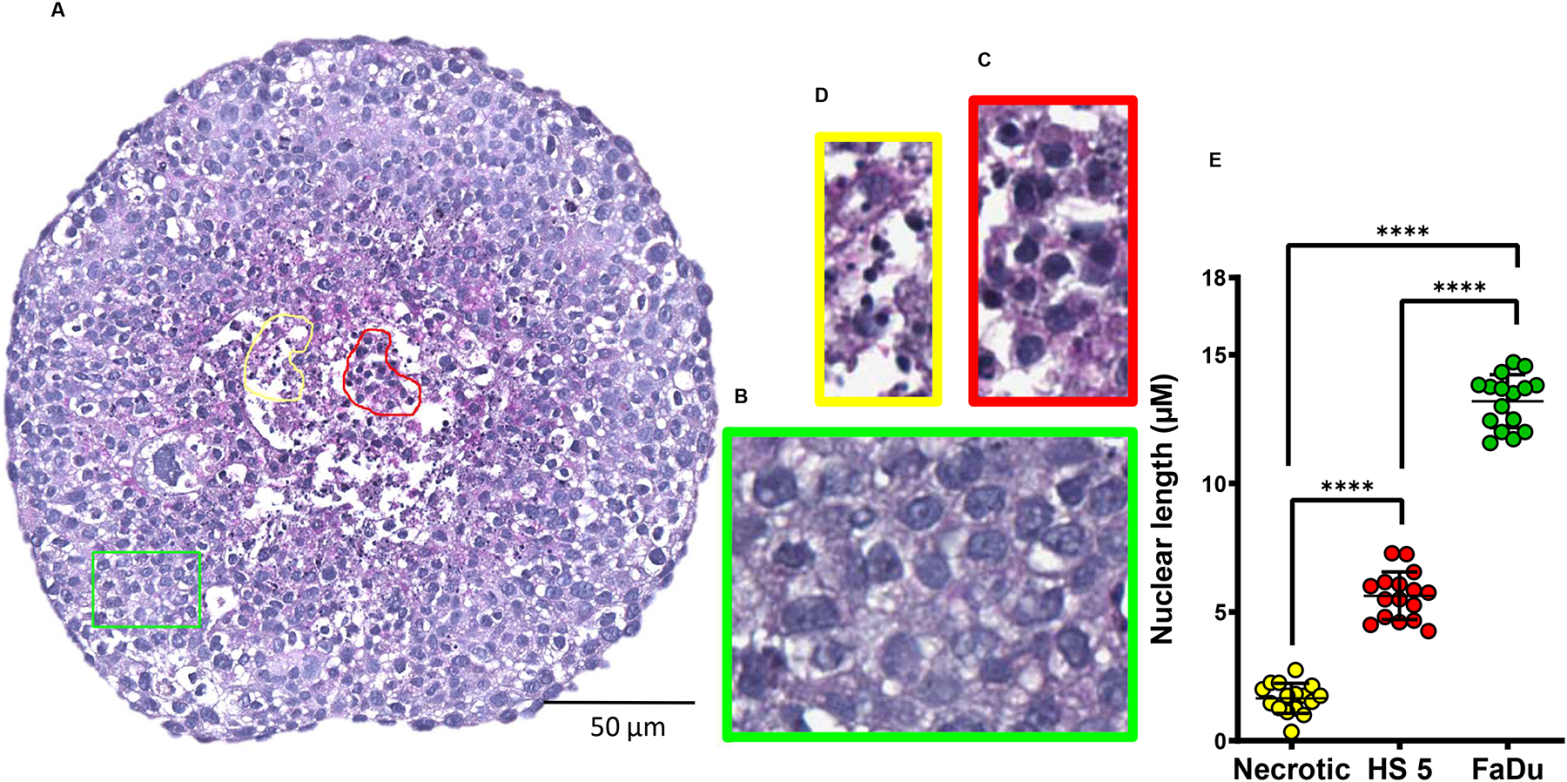
**Histological characterisation of the 3D-tumouroid co-culture.** (A) 3D-tumouroid co-culture grown for 10 days and for the histological assessment, FFPE section of the 3D-tumouroid co-culture was stained using PAS. The PAS-stained section was scanned by Leica Aperio Image scanner (representative image is at 10X objective). Morphology of different cell types present within the 3D-tumouroid co-culture were matched with the histology of the cells when grown alone (Fig. 2D). (B–D) From the whole image of the 3D-tumouroid co-culture, regions were annotated and presented separately in coloured boxes to clearly distinguish different cell types. (B) FaDu (green coloured box), (C) HS-5 (red coloured box) and (D) necrotic (yellow coloured box) cells. Representative images are at 40X objective. (E) Cells present within these coloured boxes were analysed further based on the differences in their nuclear lengths measured by Leica Image Scope software. Groups were compared in Graphpad Prism 9.5.0 software for unpaired two-tailed Welch's *t*-test. ****P<0.0001.

Next to the growth kinetics analysis, we performed the histological assessment to characterise the 3D-tumouroid co-culture (Fig. 5A–E). The histology revealed that the cells with larger nuclear sizes were at the periphery (Fig. 5A,B) and at the centre, there were two different cell types that could be differentiated based on their shorter nuclear lengths (Fig. 5A,C,D). Within the 3D-tumouroid co-culture, the FaDu and HS-5 cells were identified based on their histological characteristics when grown alone (Fig. 2D). Further, the histological features of a ‘washed-out’ appearance of the cytoplasm and formation of tiny or ‘ghost’ nuclei help to identify these cells as the necrotic cell types (Fig. 5C). A significant difference in the nuclear length of these three cell types were also identified (Fig. 5E).

**Fig. 6.**
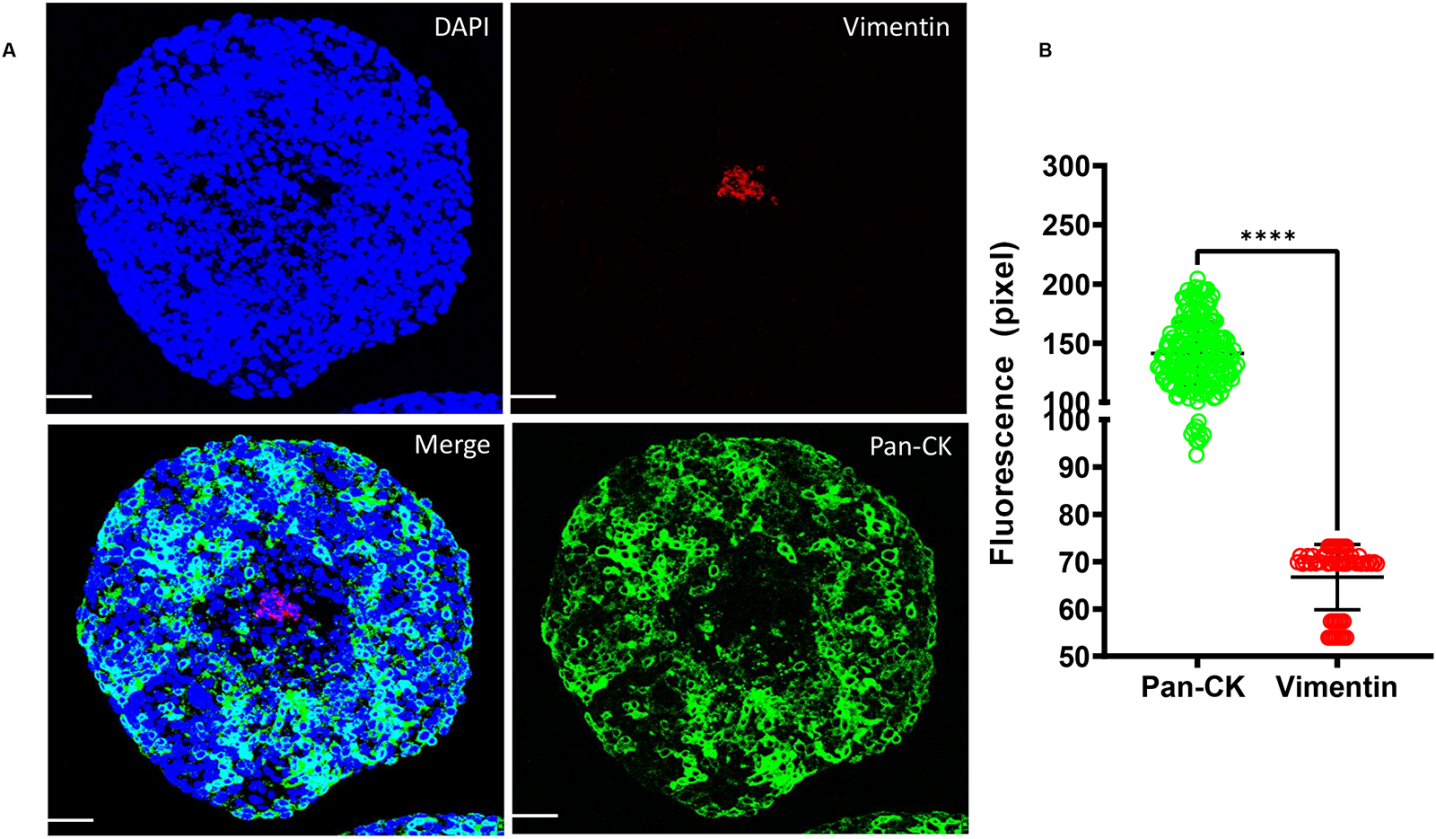
**Characterisation of the 3D-tumouroid co-culture based on the epithelial and mesenchymal biomarkers.** (A) The FFPE block of the 3D-tumouroid co-culture that was used for the histological characterisation (Fig. 5) was used again and another section was immunostained with pan-cytokeratin and vimentin as done in Fig. 2A. Composite images are pan-cytokeratin (green), vimentin (red) and nucleus (blue). Images were captured using Leica SPE confocal microscope under 10X objective with zoom factor 2 and scale bar: 20 μM. (B) Fluorescence intensity of pan-cytokeratin (green) and vimentin (red) positive cells were measured using ImageJ software and data was analysed using Graphpad Prism 9.5.0 software for unpaired two-tailed Welch's *t*-test. ****P<0.0001.

After the histological characterisation, we performed immunostaining of pan-cytokeratin and vimentin to identify the FaDu and HS-5 cells within the 3D-tumouroid co-culture (Fig. 6A), as performed previously when these two cell types were grown alone in their 3D-multicellular form (Fig. 2A). We observed vimentin-stained stromal cells were located at the centre of the 3D-tumouroid co-culture (Fig. 6A). Whereas pan-cytokeratin-stained epithelial cells were predominantly located at the periphery of this 3D-tumouroid co-culture (Fig. 6A). Fluorescence intensity of the pan-cytokeratin was ∼twofold higher than to the vimentin fluorescence intensity (Fig. 6B).

**Fig. 7.**
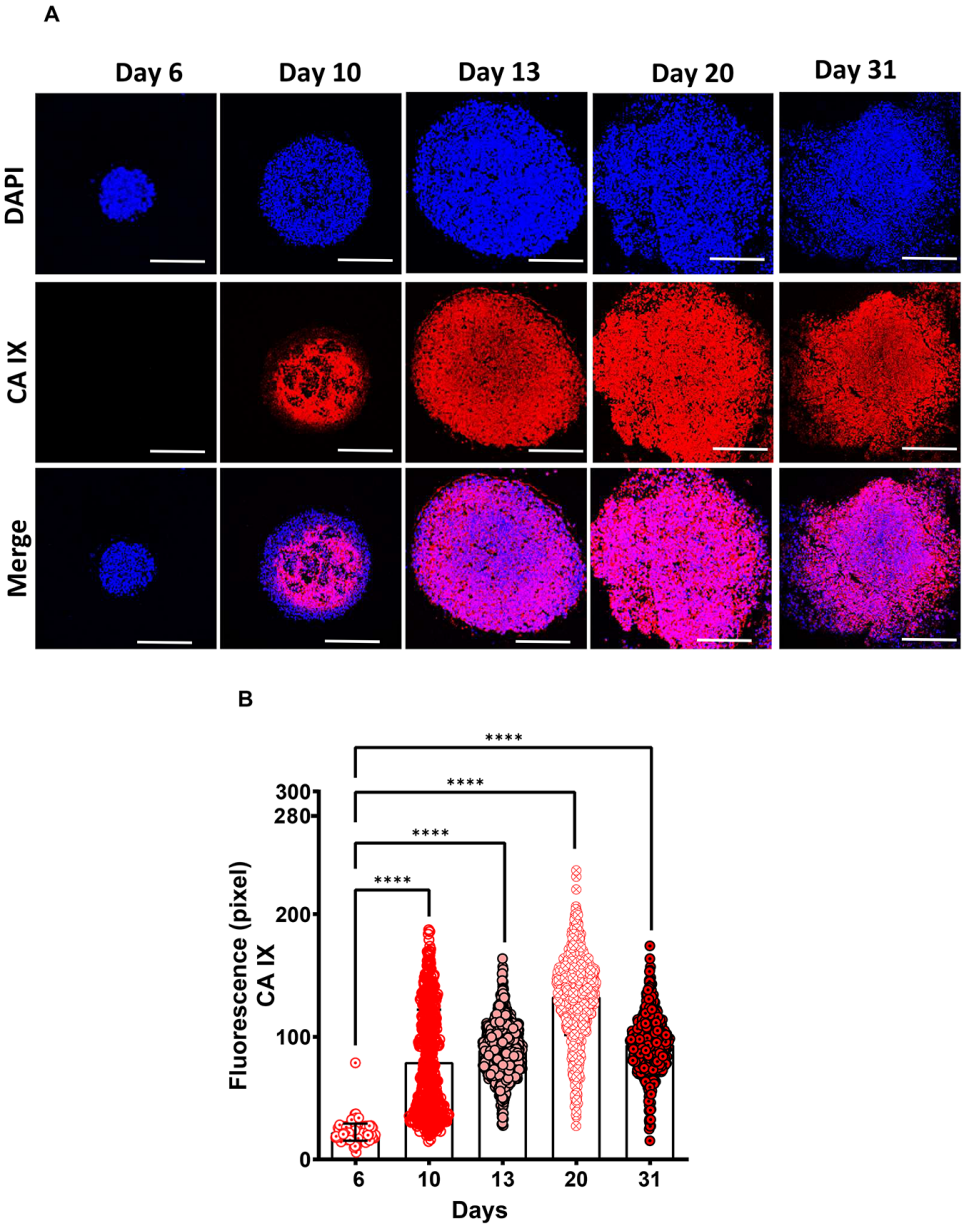
**Identification of the development of hypoxia in 3D-tumouroid co-culture.** (A) 3D-tumouroid co-cultures were grown until different time points (days: 6, 10, 13, 20 and 31) and then the FFPE blocks of these 3D-tumouroid co-cultures were prepared. From these FFPE blocks, sections were stained with CAIX to observe the progression of hypoxia. Stained images were captured using Leica SPE confocal microscope under 10X objective with zoom factor 2. Scale bar: 20 µM. Composite images are CAIX (red) and nucleus (blue) and it was observed that the hypoxic-rich environment started to develop from day 10 and persisted until day 31. (B) Fluorescence intensity of CAIX (red) positive regions were measured using ImageJ software and data were analysed using Graphpad Prism 9.5.0 software for unpaired two-tailed Welch's *t*-test. ****P<0.0001.

We have further characterised the 3D-tumouroid co-culture based on the formation of hypoxia as seen inside the TME with increasing in tumour volume (Hildingsson et al., 2022). To identify hypoxic regions, we have used CAIX biomarker (Forker et al., 2018). We measured the fluorescence intensity of CAIX for 31 days and our results show that significance increase of CAIX fluorescence intensity on day 10, 13, 20 and 31 compared to day 6 and the maximum intensity of CAIX was reached on day 20 (Fig. 7A,B).

## DISCUSSION

Approximately 15% of HNC cases are HPC types and consumption of alcohol and tobacco are the common causes of HPC (Bozec et al., 2023). It is mostly diagnosed at a late stage and is associated with a high risk of recurrence and metastasis (Bozec et al., 2023). Survival rate of HPC is poor (25–30%) (Bozec et al., 2023; Pracy et al., 2016) and despite this, there are very limited studies on HPC, which is an unmet clinical need. HPC patients are treated in a similar way to laryngeal cancer patients and thus, therapeutic management of HPC is debatable (Bozec et al., 2023). For the locally advanced tumours, primary surgery±RT is the first choice (Bozec et al., 2023; Eckel and Bradley, 2019; Pracy et al., 2016). However, very often this primary surgical treatment affects the function of patient's breathing, voice and swallowing, which has an impact on quality of life. Nevertheless, for recurrent disease, if RT fails, surgical salvage has a low success rate and restoration of larynx function is extremely challenging (Pracy et al., 2016). In some HNC patients, RT with immunotherapy has been given in a trial setting in metastatic and recurrent cases (McBride et al., 2021). However, there are few clinical trials in HPC with targeted therapy/immunotherapy in combination with RT (https://clinicaltrials.gov/ct2/show/NCT03894891). Thus, more laboratory-based research is required to understand the mechanism(s) of RT failure and to exploit new combination approaches with RT.

RT-induced immune responses are complex and can either activate or suppress the immunity and influence patient's response to therapy (Portella and Scala, 2019). RT kills both cancer and non-cancer cells within the TME (Cytlak et al., 2022). The dying cells release their DNA and damage-associated molecular patterns (DAMPs) to activate inflammatory responses (Cytlak et al., 2022). The cytosolic DNA/DAMPs are sensed by the stimulator of interferon genes (STING) and generates immune-stimulatory paracrine signals to control tumour and immune cells locally and at distant sites (Harding et al., 2017). The latter being known as an ‘abscopal effect’ (Harding et al., 2017). However, RT may have adverse effect, as well, that can reinforce activation of immune-suppressive molecules called immune checkpoints (ICPs) (Sato et al., 2017), and ICPs are immunotherapy targets (Donlon et al., 2021; Sharma et al., 2021). Thus, to understand the RT-induced complex inflammatory response, use of sophisticated models are indispensable and the 3D cell model as an alternative of replacing animals in immune-oncology research is gaining attention (Boucherit et al., 2020; Di Modugno et al., 2019; Fitzgerald et al., 2020; Neal et al., 2018). Furthermore, with the advancement of RT approaches (e.g. MRI-guided treatment, PET-containing linear accelerator, gamma knife treatment, proton beam therapy and carbon beam therapy) (Chandra et al., 2021) the mode of RT treatment in HPC has not been changed (Eckel and Bradley, 2019). Thus, to accelerate the RT research with the most advanced approaches in HPC, 3D cell models are much more convenient compared to the 2D cell culture and animal models (Doctor et al., 2020; Thiagarajan, 2021). Also, the 3D cell model has gained accepted under the guidance of American Society for Radiation Oncology for testing any new drug(s) or biomarker(s) in combination with RT before taking it to the clinic where it passes through the rationale-based early-phase clinical trials (Bristow et al., 2018). Thus, in this study, we aimed to develop a physiologically relevant 3D cell model of HPC that can be used for immuno-oncology research in near future.

There are different methods to achieve the 3D cell model (e.g. hanging drop, low adhesion plate, use of micro-structured substrates, magnetic levitation using magnetic culture, matrix or scaffold-based approach, air-liquid interface, spinner bioreactor, rotational bioreactor, vibrating bioreactor, microfluidics and 3D-bio printing) and each technique has its own advantages and limitations (Bonartsev et al., 2022; Di Modugno et al., 2019; Fitzgerald et al., 2020). Further, different researchers use the terminologies of depicting 3D cell models differently (Friedrich et al., 2007). To establish our tumour-stroma based 3D-tumouroid co-culture model, we have used the low adhesion plate. Our 3D cell model looked similar to a tumour-like structure, and thus we called it ‘tumouroid’. The use of low adhesion plate for growing cells spontaneously in a form of 3D-spheroid is based on the limitations of cell adhesion to the surface of the plates leading to the formation of cell aggregation. The Nunclon Sphera U-shaped-bottom microplates contain the surface with low adhesion/non-adhesive/micro or nanostructured properties (chemical components undisclosed by the service provider). These 96-well plates with small diameter and round-shaped bottoms allowed the enlarging of surface curvature radius and also makes the localisation of aggregate of cells in a 3D-spheroid more precise. This method is quite easy to use, cheap, reproducible, has shown good results in differentiation, multiplication and this method is adapted for many types of cancer cells including HNC (Bonartsev et al., 2022). Some researchers have also reported the disadvantages of this method as they have found some tumour cells do not form stable aggregate in a 3D-spheroid structure (Bonartsev et al., 2022). However, we have ruled out this at the beginning by growing FaDu and HS-5 cells independently in a multicellular structure as 3D-tumouroids (Fig. 2A,D) and later adapted this method to establish our 3D-tumouroid co-culture model (Fig. 4A).

While there is no particular study on the role of the tumour-stroma ratio in HPC types but, overall, the tumour-stroma ratio within the TME plays an important role in prognosis and survival of HNC patients (Almangush et al., 2021). Stromal rich TME is responsible for poorer absorption of drugs (Van Zundert et al., 2020). Nevertheless, a stromal rich TME is also responsible for radio resistance (Krisnawan et al., 2020) and modulates the RT effect for immunosuppressive responses (Berg and Pietras, 2022; Donlon et al., 2021; Menon et al., 2019). The well-known mechanisms of stromal cell mediated immunosuppression are due to upregulation of immunosuppressive molecules or so-called ICPs including Indoleamine 2,3-dioxygenase 1 (IDO1), Programmed death-ligand 1 (PD-L1) etc. (English, 2013; Krampera, 2011). As mentioned previously, ICPs are immunotherapy targets (Donlon et al., 2021; Sharma et al., 2021) and thus, understanding the tumour-stroma interaction is pertinent in 3D cell model of HPC.

Adding artificial matrix components such as Matrigel has been shown to influence the epithelial–mesenchymal transition (EMT) of HNC cells (Dal Vechio et al., 2011). Thus, we have avoided any external factors that could influence the TME. In our study, we observed the pan-cytokeratin and vimentin immunostaining remained unaltered in the 2D and 3D cell culture condition of FaDu and HS-5 cells when grown alone (Figs 1B, 2A). This further justifies the benefit of not adding Matrigel in our protocol and later we combined FaDu and HS-5 cells together to recapitulate a physiologically relevant tumour-stroma based 3D-tumouroid co-culture model (Fig. 4A).

Comparison of growth kinetics revealed, significant increase of tumouroid growth by adding stromal cells (HS-5) to the cancer cells (FaDu) (Fig. 4A,B). This may be due to the role of chemokines (e.g. CXCL7) secreted by the HS-5 stromal cells as a growth promoting factors (Windus et al., 2014). The use of HS-5 as stromal cells to establish tumour-stroma based co-culture model has been studied in other cancer types. These include, prostate cancer (cell line: PC-3) (Windus et al., 2014), leukemia (cell lines: K-562, LAMA-84, HL-60, Kasumi-1, Nalm-6, KG1a, NKTert, OCI-AML3, and primary cancer cells) (Garrido et al., 2001; Lu et al., 2019; Podszywalow-Bartnicka et al., 2018; Usuludin et al., 2012) and multiple myeloma (cell lines: MM1.S, U266, RPMI.8226, MMCL, U266, OPM-2) (Belloni et al., 2018; Waldschmidt et al., 2022) but, to our knowledge, never studied in HPC or in combination with FaDu cells. Similarly, FaDu cells as a model of HC types were studied in a co-culture model with different cell types like; cancer-associated fibroblasts (CAFs) (Fík et al., 2014; Gehrke et al., 2016; Kolar et al., 2012; Yakavets et al., 2019; Young et al., 2018) and primary mesenchymal stromal cells (MSCs) (Ruhle et al., 2022) but, to our knowledge, never studied in combination with HS-5 cells. Another reason for selecting HS-5 cells in our study is the presence of immunological signatures (e.g. CD54, HLA-ABC, HLA-DR, CD274, Fas, FasL etc.) and immunosuppressive activity (inhibitory effect on T cell proliferation) in this cell type (Adamo et al., 2020). These appear to recapitulate our 3D-tumouroid co-culture model as a ‘cold tumour’ types (Too et al., 2021). However, further studies would be required to prove that. We have observed the localisation of vimentin positive HS-5 cells at the centre of the 3D-tumouroid (Fig. 6A) and similar phenomena was also observed by another researcher (Yakavets et al., 2019). However, the underlying reason is unknown and needs further study. Moreover, the histology of the 3D-tumouroid co-culture clarified morphology of these stromal cells is different from the necrotic cells (Fig. 5).

Hypoxia is one of the major contributing factors for the development of radio-resistant tumours. Inside the TME, abnormal blood vasculature causes alterations in oxygen supply to tumour cells generating hypoxic regions. Depending on oxygen tension, severity of hypoxia can be classified as mild (1–3% O2), moderate (0.1–1% O2) and acute (<0.1% O2). Chronic hypoxia is characterised by the persistence of an acute hypoxic condition for prolonged period distant from blood vessels (∼100–200 μm) and results in the formation of an anoxic core. Oxygen tension is also associated with reduction in the gradient of nutrient, lactate and pH, which can influence transition from cell proliferation (near oxic zone or blood vessel) to cell necrosis (near anoxic regions) (Leek et al., 2016). Hypoxic tumour cells (<0.1% O2) are ∼three times less radiosensitive compared to the well-oxygenated proliferative tumour cells. Furthermore, hypoxia drives the immunosuppression in TME by multiple mechanisms (e.g. immunosuppressive metabolites) (Fu et al., 2021; Li et al., 2018; Vito et al., 2020). Therefore, there is an urgent need to elucidate the underlying mechanism of development of radioresistant tumours and strategies to target hypoxic tumour cells. To achieve that it is relevant to characterise hypoxic zones within the 3D-tumouroid co-culture model.

Direct measurement of hypoxia using oxygen electrodes is impractical, and despite advances in hypoxia imaging methods none are in routine clinical practice (Fleming et al., 2015; Hammond et al., 2014). Thus, identifying robust endogenous biomarker(s) of tumour hypoxia that can potentially identify HC patients of high-risk group is clinically crucial. High expression of the hypoxia marker CAIX in soft tissue sarcoma was found to be associated with poor survival in the Phase III adjuvant RT VorteX trial but two other hypoxia markers Hif-1a and GLUT1 were not (Forker et al., 2018). Use of CAIX as a hypoxic biomarker in 3D *in vitro* model as well as in *in vivo* model is reported (McIntyre et al., 2012). Immunohistochemical analysis of high CAIX expression in T3 and T4 hypopharyngeal cancer was also found to be an adverse prognostic factor for disease-specific survival (Bernstein et al., 2015). Thus, we have used CAIX, which has been previously shown to be a superior surrogate imaging marker for tumour hypoxia (Tafreshi et al., 2016). Hypoxia and large tumour volume are negative prognostic factors for patients and the knowledge of progression of hypoxia can be important to determine individualised RT treatment strategy (Hildingsson et al., 2022). Thus, in our study we introduced a time point based study by developing the 3D-tumouroid co-cultures and measuring the progression of hypoxic regions with time to mimic the potential clinical scenario.

### Conclusion

Unlike the conventional platinum-based chemo-radiotherapy (CRT) treatment from the UK's standard guidelines of CRT in HNC (Nutting, 2016) or HPC (Pracy et al., 2016), which shows variable response, our innovative research tool will be allowing rationale-based testing of new classes of radiosensitisers and immunotherapy as a novel combination with the most advanced RT approaches (e.g. proton/carbon beam therapy). These novel therapy combinations could provide treatment benefit to those patients who cannot receive platinum drugs with RT due to renal inefficiency and or have unresectable tumours receiving only RT with potentially poor survival.

### Future directions

The DNA damage repair inhibitors (DDRi) with RT can be used as radiosensitisers to increase DNA damage-induced cell killing (McLaughlin et al., 2020) (Fig. 3; Fig. S2), through the release of cytosolic DNA/DAMPs and STING activation (Reislander et al., 2020). Thus, our perception is that RT +/– DDRi or STING agonists may increase cell death and release cytosolic DNA/DAMPs to activate innate immune signalling. We have tested our hypothesis in the 2D cell-culture model (Fig. S4). However, whether the same observation can be replicated within our 3D-tumouroid co-culture model needs further investigation. If successful, then that would bring us lead to a step closer to our long-term aim of using RT with DDRi or STING agonists and immunotherapy in HNC particularly in HPC type.

Another impact of our 3D-tumouroid co-culture model is to use that as a new ‘pathology tool’. The time of starting treatment from the diagnosis of HNC/HPC is ∼30 days (Yao et al., 2021). Within the progressive tumour the molecular alterations take place very rapidly and the longer diagnosis time has an impact on therapy failure. Although the patient-derived xenograft tumour model can be used for the clinical decision-making it has some limitations including low engraftment rate of tumour cells in mice and longer time of drug screening (4–8 months). Thus, our long-term goal is to exploit patient-derived tumouroids as a new ‘pathology tool’ that can help in more rapid decision making.

## MATERIALS AND METHODS

### Cell lines and culture conditions

A bone stromal cell line HS-5 was recovered from the cell bank of Centre for Cancer at the Paul O'Gorman Building, Newcastle University and FaDu cell was gifted from Catharine West, University of Manchester, UK. Cells were mycoplasma tested (MycoAlert, Lonza, Basel, Switzerland) every 2 months and authenticated by DNA profiling. Cells were routinely maintained in Dulbecco's Modified Eagle Medium (DMEM; Sigma D5796) containing L-Glutamine, 10% fetal bovine serum (FBS; Gibco), and 1% Penicillin/Streptomycin (Sigma P4333) in T75 flasks (Corning) at 37°C, 5% CO2 in a humidified incubator (SANYO MCO-20AIC). Exponentially growing cells were maintained at 80% confluency and regularly passaged at 1:5 to 1:10 cell dilutions using 1X trypsin (Sigma T4174).

### Image flow cytometry

Cells were characterised for their epithelial and non-epithelial features. For this characterisation, imaging flow cytometry was done probing the cells with Alexa Fluor 488 anti-pan cytokeratin (ab277270, Abcam) and Alexa Fluor 647 anti-vimentin (ab194719, Abcam) antibodies. Cells growing in monolayer were washed once with 1X PBS and dissociated using 1X trypsin. The trypsin was neutralised using complete medium followed by washing once with 1X PBS. Cells were then fixed with 80% methanol for 5 min. Methanol was added dropwise while vortexing the cells for the homogeneous fixation. Next, cells were washed once with 1X PBS and then permeabilised with 0.1% PBS-Triton X-100 (Sigma T8787) for 15 min. After permeabilisation, cells were washed once again with 1X PBS and then incubated in 10% goat serum (Sigma G9023) for 1 h to block non-specific protein–protein interaction. After blocking, cells were incubated with the antibodies (1:500 dilution) for 1 h before the imaging flow cytometry. Images of 5000 cells from each of the staining conditions were captured on the ImageStreamX Mark II with fluorochrome excitation at 388 and 562 nm as well as brightfield illumination. Images of the objects were gated by plotting the aspect ratio of the objects against the area of the brightfield images to select round single objects. Images in focus were subsequently gated according to a threshold of the ‘gradient root mean square’ parameter. All in focus round single cells were plotted in an XY plot of cytokeratin fluorescence intensity against vimentin fluorescence intensity to identify the relative degree of epithelial and non-epithelial phenotypes.

### 3D-tumouroid cell culture

3D-tumouroids were developed by growing cells in 96-well plates (Nunclon Sphera U-shaped-bottom microplates, Thermo Scientific). During the cell seeding in the 96-well plates, medium from the T75 flasks was aspirated, the cells were washed once in 1X PBS and dissociated using 1X trypsin. The trypsin was neutralised using complete medium. Cell viability was checked by trypan blue staining (Sigma T8154) followed by cell counting using hemocytometer cell counter (Neubauer Improved Counting Chamber, Hawksley). Stock cells with >90% viability were taken for the tumouroid formation. The stock cells were diluted in complete medium to make calculations for cell seeding density easier (e.g. 100 µl from the 10^4^/ml dilution is equal to 1000 cells). The final volume was maintained at maximum 300 μl in each well of the 96-well plates. Media was changed on alternate days to prevent any acidosis. Media aspiration was done using manual pipetting and care was taken to keep the growing cells untouched by the sharp pipette tips. 3D-tumouroid co-cultures were developed by simultaneous seeding of FaDu and HS-5 cells at 1:1 ratio. Brightfield images of the tumouroids as well as the tumouroid co-cultures were captured using EVOS XL microscope and the diameters were analysed using ImageJ software. The tumouroids were spherical in shape and thus the volume of the tumouroid was analysed following the formula V=4/3 πr³ (V, tumouroid volume; r, radius).

### Formalin-fixed and paraffin embedded (FFPE) tumouroids

For preparing the FFPE tumouroids, at suitable end points, as much media as possible was aspirated from each well of the 96-well plates without disturbing the tumouroids. Tumouroids were rinsed with 1X PBS followed by fixation with 4% paraformaldehyde (PFA, Sigma 16005) for 30 min to 1 h. After fixation, PFA was removed and tumouroids were washed again with 1X PBS. The next step was transferring the tumouroids to HistoGel (Eperdia, HG-4000-012) embedding medium. Histogel was melted and kept at 65°C using a heat block until use. A small amount of Histogel was set into a microcentrifuge tube cap, used as a mould. Tumouroids from each well of the 96-well plates were transferred using wide bore pipet tips onto the Histogel. Excess liquid was removed and tumouroids were covered with few more drops of molten HistoGel and then transferred on a cold surface (e.g. ice) for 2–5 min for rapid solidification. Care was taken to ensure that tumouroids were placed at the centre of the blocks. Solidified HistoGel containing tumouroids were transferred from the moulds into labelled processing cassettes and then placed in an automated tissue processor (Leica Tissue Processor) for processing (ethanol 70%, 95% x2, 100% x3; xylene x4, 2× molten wax; total time 13 h). After the processing step, cassettes were embedded using an embeddor (Microm EC 350-1). Embedded samples were then transferred to cold plate (Microm EC 350-2). Once embedded, tumouroids samples were sectioned (4 µM) using a microtome (Microm HM 315) and placed onto SuperFrost Plus slides (VWR, 631-0446) for histology and immunostaining.

### Histology of the tumouroids

For histology, periodic acid-schiff (PAS) staining was done. PAS staining can differentiate cellular morphologies and cellular components (e.g. glycogen, mucopolysaccharides, basement membrane, colloid materials etc). PAS positive substances stain pink to red and nuclei stain blue due to counterstaining with Hematoxylin. For the staining, standard procedure of the PAS kit (Sigma 395B) was followed using the FFPE tumouroid sections. Briefly, sections of the tumouroids on glass slides were deparaffinised and hydrated using deionised water. Slides were then immersed in ‘periodic acid’ solution for 5 min at room temperature (18–26°C). This, followed by rinsing the slides in several changes with distilled water. Slides were then immersed in ‘Schiff's reagent’ for 15 min at room temperature (18–26°C). Next, slides were washed in hot running tap water for 5 min. Counterstaining was done using Hematoxylin solution for 90 s. Next, slides were again rinsed in running tap water and finally, slides were dehydrated and the tumouroid sections were mounted in mounting media. Stained sections were scanned using Leica Aperio Image Scanner.

### Immunostaining of the tumouroids

For the immunostaining, tumouroids on glass slides were dewaxed and hydrated using deionised water. Next, antigen retrieval was done using a pressure cooker in citrate buffer (pH6). For blocking endogenous peroxidase, slides were immersed in 3% H_2_O_2_ solution in Tris buffered saline (TBS) [Tris(hydroxymethyl)aminomethanol, Sigma 102362011; sodium chloride, Fisher 7647-14-5; HCl, Fisher H/11/PB15] of pH7.6 for 15 min. Slides were then washed in Tris buffered saline with Tween 20, Sigma P1379 (TBST) for 2×10 min. Washing was followed by blocking for 1 h with the blocking buffer [2% BSA, Sigma A2153; 10% (w/v) milk powder; 10% goat serum, Sigma G9023 in TBST] at room temperature. After this blocking step, conjugated or unconjugated primary antibodies were added and incubated at 4°C overnight. Next day, the slides were washed for 3×10 min in TBST. For the unconjugated primary antibodies, next step was the incubation with the secondary antibodies for 1 h at room temperature followed by washing in TBST for 3×10 min. Then, the slides were stained with DAPI (1:1000; Sigma D9542) at room temperature. Where conjugated primary antibodies were used, adding the secondary antibody step was omitted. Slides were washed once for 10 min in TBST and then coverslips were mounted using anti-fade mountant (Invitrogen, prolong glass antifade mountant). For the characterisation of different cell types, Alexa Fluor 488 Anti-pan-cytokeratin (ab277270, Abcam) and Alexa Fluor 647 Anti-vimentin (ab194719, Abcam) conjugated antibodies (1:500 dilution) were used (the same antibodies used for the image flow cytometry). For the identification of hypoxic cells, CA-IX (1:200 dilution; sc-365900, Santa Cruz) with Alexa Fluor 546 (1:1000 dilution; Invitrogen) secondary antibodies were used. Stained slides were observed under the Leica SPE confocal microscope.

### Image analysis

Immunofluorescence images of the tumouroids were quantified using ImageJ software. Nuclear length of the PAS-stained cells was measured using Leica Image Scope software. Analysis of morphological features from the fluorescence images were done using MorphoLibJ plugin with ImageJ software (Legland et al., 2016) and morphological segmentation of the nuclei was done following the steps of pre-processing, thresholding and cleaning-up. All statistical analysis was performed using Graphpad Prism 9.5.0 and the following *P*-value cut-offs were considered: *P-value<0.05; **P-value<0.01; ***P-value<0.001 and ****P-value<0.0001.

## Supplementary Material

10.1242/biolopen.059949_sup1 Supplementary information
