# Supplementary material for: Bioengineering of a tumour-stroma 3D-tumouroid co-culture model of hypopharyngeal cancer
Source: Biol Open. 2023 May 17;12(5):bio059949. doi: 10.1242/bio.059949 (PMC10214853; doi:10.1242/bio.059949)
Supplement: Supplementary information [file biolopen-12-059949-s1.pdf]

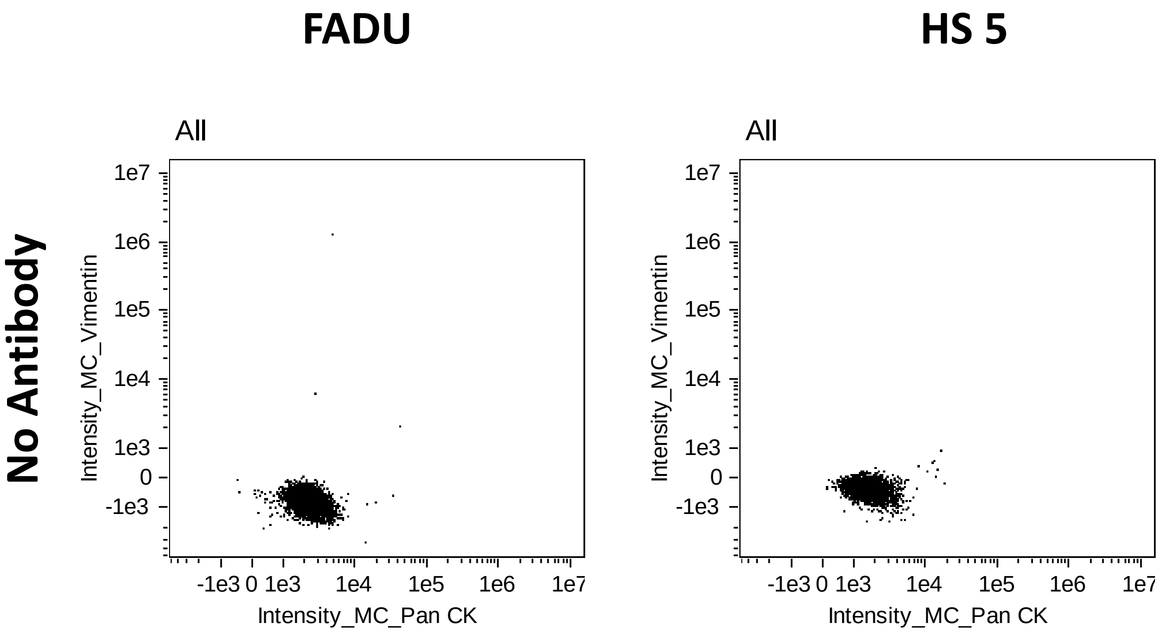


**Fig. S1. No antibody control of FaDu and HS 5 cells during characterisation of these tumour (FaDu) and stromal (HS-5) cells by image flow cytometry.** FaDu and HS 5 cells without any antibodies were used as the control while acquiring the image flow cytometry data to characterise these two cells for their epithelial and mesenchymal features. Instrument used, number of cells sorted, fluorescent excitation/emission, image acquisition and gating strategies were the same as mentioned in Fig.1 B and C.

Biology Open • Supplementary information

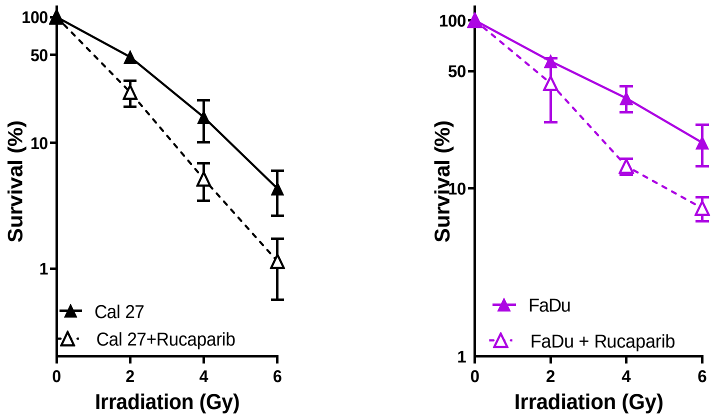


| Cell lines | | Cal 27 | FaDu |
|---|---|---|---|
| LC50 (Gy) | RT | 2.5 ± 0.23 | 3.2 ± 0.42 |
| | RT + Rucaparib | 1.9 ± 0.22 | 2.4 ± 0.55 |
| Sensitisation at LC50: | | 1.3 P= 0.0347 (*) | 1.3 p = 0.1010 |
| 2 Gy (% Survival) | RT | 48.2 ± 0.04 | 57.2 ± 0.027 |
| | RT + Rucaparib | 25.3 ± 0.1 | 42.2 ± 0.30 |
| Sensitisation at 2 Gy: | | 1.9 P= 0.0441 (*) | 1.3 p = 0.4823 |
| 4 Gy (% Survival) | RT | 16.0 ± 0.1 | 34.4 ± 0.105 |
| | RT + Rucaparib | 5.2 ± 0.02 | 13.6 ± 0.026 |
| Sensitisation at 4 Gy | | 3.0 P= 0.1994 | 2.5 p = 0.0678 |
| 6 Gy (% Survival) | RT | 4.3 ± 0.02 | 18.7 ± 0.089 |
| | RT + Rucaparib | 1.1 ± 0.01 | 7.6 ± 0.021 |
| Sensitisation at 6 Gy | | 3.9 P= 0.1919 | 2.5 p = 0.1588 |

**Fig. S2. Radiosensitisation by the DNA damage response inhibitor (PARP inhibitor).** Survival of head and neck cancer cells (Cal 27 and FaDu) following exposure to 1µM concentration of the PARP inhibitor rucaparib ± increasing doses (0-6 Gy) of ionising radiation (IR) for 24 h and survival was determined by clonogenic assay. Survival in the table is given as a percentage relative to the survival in 0.5% DMSO only as the control. Sensitisation factor is the ratio between survival at any fixed dose of IR in the absence and presence of a rucaparib. Data are mean ± SEM (N=3). LC50 values defined as the dose (IR) where survival was 50% were measured by interpolation of the survival vs dose curves using GraphPad Prism software. Treated groups were compared using unpaired t-test and * represents $p < 0.05$.

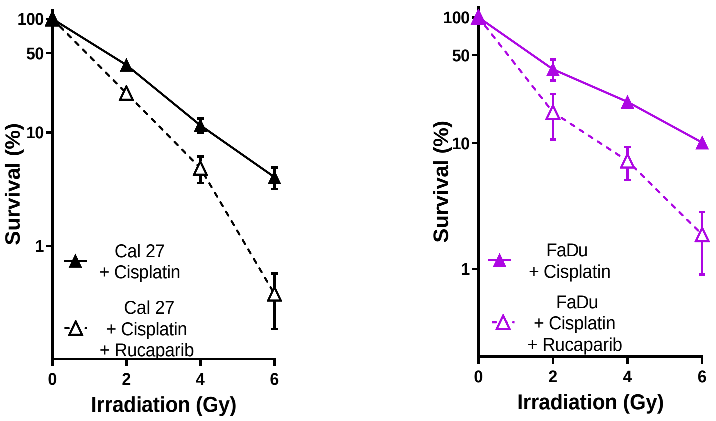


| Cell lines | | Cal 27 | FaDu |
|---|---|---|---|
| **LC50 (Gy)** | RT | 2.3 ± 0.07 | 2.5 ± 0.29 |
| | CRT + Rucaparib | 1.9 ± 0.04 | 1.8 ± 0.28 |
| **Sensitisation at LC50** | | 1.2 p= 0.0030 (**) | 1.4 p= 0.0462 (*) |
| **2 Gy (% Survival)** | RT | 39.3 ± 0.04 | 38.7 ± 0.12 |
| | CRT + Rucaparib | 22.2 ± 0.03 | 17.6 ± 0.12 |
| **Sensitisation at 2 Gy** | | 1.8 p= 0.0070 (**) | 2.2 p= 0.1041 |
| **4 Gy (% Survival)** | RT | 11.5 ± 0.02 | 21.3 ± 0.03 |
| | CRT + Rucaparib | 4.9 ± 0.02 | 7.2 ± 0.03 |
| **Sensitisation at 4 Gy** | | 2.3 p= 0.0374 (*) | 3 p= 0.0086 (**) |
| **6 Gy (% Survival)** | RT | 4.0 ± 0.01 | 10.1 ± 0.01 |
| | CRT + Rucaparib | 0.4 ± 0.003 | 1.9 ± 0.01 |
| **Sensitisation at 6 Gy** | | 10.0 p= 0.0450 (*) | 5.3 p=0.0033 (**) |

**Fig. S3. Chemo-radiosensitisation by the DNA damage response inhibitor (PARP inhibitor).** Sensitisation of chemo-radiation by rucaparib in head and neck cancer cells (Cal 27 and FaDu). Survival of cells following exposure to chemo-radiation alone (continuous line) and in combination with 1 µM rucaparib (dashed line). Cells were exposed to increasing doses of radiation (0-6 Gy) and 0.1 µM cisplatin, with or without addition of 1µM rucaparib for 24 h and survival was determined by clonogenic assay. For the chemo-radiation alone data were normalised to DMSO and for combinations they were normalised to cisplatin. Data represents means ± SEM (N=3). In the table, LC50 values defined as the dose (IR) where survival was 50%. Treated groups were compared using unpaired t-test using GraphPad Prism software. * and ** represent $p < 0.05$ and $p < 0.01$ respectively.

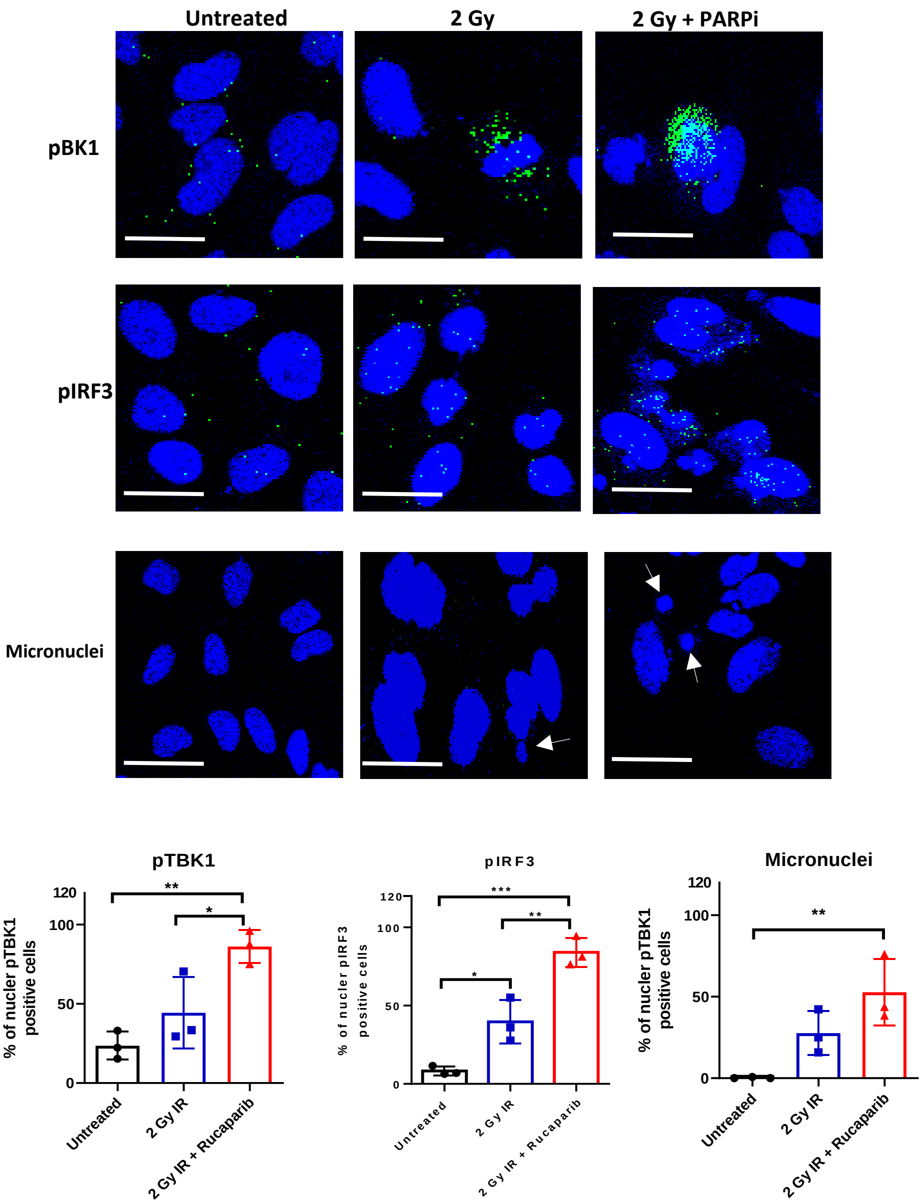


**Fig. S4. Combination of ionising radiation (IR) and PARP inhibitor (PARPi) rucaparib induces STING-mediated innate immune activation.** As a proof-of-the-concept, HeLa cells were plated on coverslips and allowed to adhere for at least 12 h prior to treatment with 2 Gy IR ± 1μM rucaparib (72 hr). Immunofluorescence (IF) staining of pTBK1 (green), pIRF3 (Green) and Micronuclei (white arrow) were the indications for STING-mediated innate immune activation. Nucleus (blue). Groups were compared using unpaired t-test using GraphPad Prism software. * , ** and *** represent $p < 0.05$, $p < 0.01$ and $p < 0.001$ respectively. Scale bar is 20μM.

Biology Open • Supplementary information

For the IR staining, Cells were fixed with ice-cold methanol for at least 30 min before staining. Coverslips were washed in phosphate-buffered saline (PBS) containing 0.5% Triton X-100 (PBS-T) for 3 X 10 min. Washing was followed by blocking for 1 h with the blocking buffer (2% BSA, Sigma A2153; 10% (w/v) milk powder; 10% goat serum, Sigma G9023 in TBS-T) at room temperature. After the blocking step, primary antibodies were  added and incubated at 4° C overnight. The coverslips were subsequently washed 3 X 10 min in PBS-T followed by incubation with the secondary antibody; Goat Anti-Rabbit IgG H&L (Alexa Fluor® 488) preadsorbed (ab150081) (1:1000 dilution) for 1 h at room temperature and then washed 3 X 10 min in PBS-T. Then, the coverslips were stained with DAPI (1: 1000; Sigma D9542) for 30 min at room temperature. Finally, the coverslips were mounted using anti-fade mountant (Invitrogen, prolong glass antifade mountant). Stained coverslips were observed under the Leica SPE confocal microscope and the images were analysed using ImageJ software. Primary antibodies used and their working dilutions were as follows. Phospho-IRF-3 (Ser396) (D6O1M) Rabbit mAb #29047 (1:200 dilution) and Phospho-TBK1/NAK (Ser172) (D52C2) XP® Rabbit mAb #5483 (1:100 dilution).

Biology Open • Supplementary information
